# Theoretical Study on Carrier Mobility of Hydrogenated Graphene/Hexagonal Boron-Nitride Heterobilayer

**DOI:** 10.1186/s11671-018-2780-2

**Published:** 2018-11-22

**Authors:** Zhenqiang Ye, Hua Geng, Xiaoping Zheng

**Affiliations:** Department of Automation, Tsinghua University, Beijing, 100084 People’s Republic of China

**Keywords:** Hydrogenated graphene, Hexagonal boron nitride, Effective mass, Carrier mobility

## Abstract

Hydrogenated graphene (HG)/hexagonal boron nitride (h-BN) heterobilayer is an ideal structure for the high-performance field effect transistor. In this paper, the carrier mobilities of HG/h-BN heterobilayer are investigated based on the first-principles calculations by considering the influence of stacking pattern between HG and h-BN, hydrogen coverage and hydrogenation pattern. With the same hydrogenation pattern, the electron mobility monotonously decreases when the hydrogen coverage increases. With the same hydrogen coverage, different hydrogenation patterns lead to significant changes of mobility. For 25% and 6.25% HGs, the μ_e_ (ΓK) of 25% pattern I is 8985.85 cm^2^/(V s) and of 6.25% pattern I is 23,470.98 cm^2^/(V s), which are much higher than other patterns. Meanwhile, the h-BN substrate affects the hole mobilities significantly, but it has limit influences on the electron mobilities. The hole mobilities of stacking patterns I and II are close to that of HG monolayer, but much lower than that of stacking patterns III and IV.

## Introduction

Hydrogenated graphene (HG) [1, 2] is one of the most promising graphene-based materials. It has aroused widespread attention due to its extensive applications, such as hydrogen storage [3], ferromagnetism [4], fluorescence [5], and thermal rectification [6]. In contrast to metallic graphene, HG is predicted to be the semiconductor with a tunable band gap [7, 8]. Thus, it can be used as the channel material of field-effect transistor (FET) [9]. Excellent FETs should have an ultra-high carrier mobility of the channel material. As is well known, the traditional SiO_2_ substrate has a significant negative effect on FET performance [10]. Recently, the studies show that monolayer hexagonal boron nitride (h-BN) [11, 12] is a promising candidate for the substrate of graphene-based FET. Monolayer h-BN and HG are lattice-matched structures, indicating a better contact performance. Hence, HG/h-BN heterobilayer is an ideal structure of the FET’s channel. Unfortunately, there are only a few related studies about the electronic properties of HG/h-BN heterobilayer structure. The carrier mobility performance of HG/h-BN heterobilayer is still an open question.

Most of the current studies on HG are devoted to engineering the desired electronic properties via hydrogenation [13–18]. Gao et al. [13] studied the hydrogen coverage and configuration dependence of the band gap of HG. Sahin et al. [14] compared the effect of adatom-patterned (hydrogenation) and hole-patterned (removal of carbon atom) graphene nanomeshes on band structure. Shkrebtii et al. [15] investigated the band structure of HG, where the structure of HG is limited in C_16_H_n_ system (*n* = 0,2,8,16). Song et al. [16] calculated the band gap of HGs with different hexagon vacancies. Bruzzone et al. [17] calculated the mobilities of HG with different hydrogen coverage (100%, 75%, 25%) by ab-initio simulations and found 25% HG got the highest mobility. There are also some studies about applying the hydrogenation in h-BN. Chen et al. [19] utilized the hydrogenation to realize semiconductor to metal transition in h-BN. Liang et al. [20] studied the interactions between 100% HG and 100% hydrogenated h-BN. It shows that the electron mobility of HG/hydrogenated h-BN is only 50 cm^2^/(V s) which is far away from that of graphene.

In a word, the current studies on the carrier mobility of HG/h-BN heterobilayer are still not enough. The main factors which affect the carrier mobility of HG/h-BN heterobilayer, namely hydrogen coverage, hydrogenation pattern and the stacking pattern between HG and h-BN, should be clarified. In this paper, the carrier mobilities of HG/h-BN heterobilayer structures were investigated based on the first-principles calculations. Firstly, the effect of the h-BN substrate on the mobilities of HG was investigated. Secondly, the electronic properties of HG with different hydrogen coverage were compared. Finally, different hydrogenation patterns were applied in 25% and 6.25% HG to reveal the influence of hydrogenation pattern.

## Methods

All calculations were implemented in Atomistix ToolKit (ATK) [21] based on the density functional theory (DFT). The exchange correlation is the generalized gradient approximation (GGA) with the Perdew-Burke-Ernzerhof (PBE) functional. Van der Waals (vdW) correction adopted Grimme DFT-D2 method [22] for the heterobilayer structures. The cell length in *z* direction (perpendicular to the HG plane) is 20 Å, in order to eliminate the effect of its periodic images. The k-point sampling is 33 × 33 × 1 Monkhorst-Pack grid.

Deformation potential approximation (DPA) method [23] is used to investigate the carrier mobility; the expression of the carrier mobility of 2D material [24, 25] is:

1 $$ \mu =\frac{e{\mathrm{\hslash}}^3{C}_{2\mathrm{D}}}{k_{\mathrm{B}}{Tm}^{\ast }{m}_{\mathrm{d}}{E}_1^2}, $$

where *e* is the electron charge, *ћ* is reduced Planck constant, *k*_B_ is Boltzmann constant, *T* is the temperature (it is set to be 300 K in the cases), and *C*_2D_ is the elastic modulus of the propagation direction. *E*_1_ is the deformation potential constant defined by *E*_1_ = Δ*V*/(Δ*l*/*l*_0_). Δ*V* is the energy change under proper cell compression and dilatation. The change of the conduction band minimum (CBM) is used for electrons and the valence band maximum (VBM) for holes. *l*_0_ is the lattice length in the transport direction and Δ*l* is its deformation (Δ*l*/*l*_0_ is set to be − 0.01, − 0.005, 0, 0.005, 0.01). m^*^ is the effective mass in the transport direction, calculated by:

2 $$ {m}^{\ast }={\mathrm{\hslash}}^2{\left[\frac{\partial^2E(k)}{\partial {k}^2}\right]}^{\hbox{-} 1}, $$

where *k* is the wave vector and *E* is the energy. *m*_d_ is the equivalent density-of-state mass defined as *m*_d_ = (*m*_*x*_*m*_*y*_)^0.5^. Deformation potential constant and effective mass can be deduced from band structures, while the elastic modulus is extracted from phonon dispersion relations. It should be emphasized that the DPA method may overestimate the mobilities of arsenene, antimonene [26], and silicene [27] because it does not consider the effect of flexural acoustic (ZA) phonons. Shuai et al. [28, 29] discussed the applicability of DPA and found that it can estimate the electronic properties of graphene and graphyne well. The ZA phonons play a minor role in electron-phonon interactions for two-dimensional carbon materials. The electronic mobility of graphene [28] at room temperature is estimated to be 3.4 × 10^5^ cm^2^/(V s) by DPA method and 3.2 × 10^5^ cm^2^/(V s) [28] by considering all the electron-phonon interactions. As for HG, we will reanalyze the effect of ZA phonons in the next part.

## Results and Discussion

**Fig. 1:**
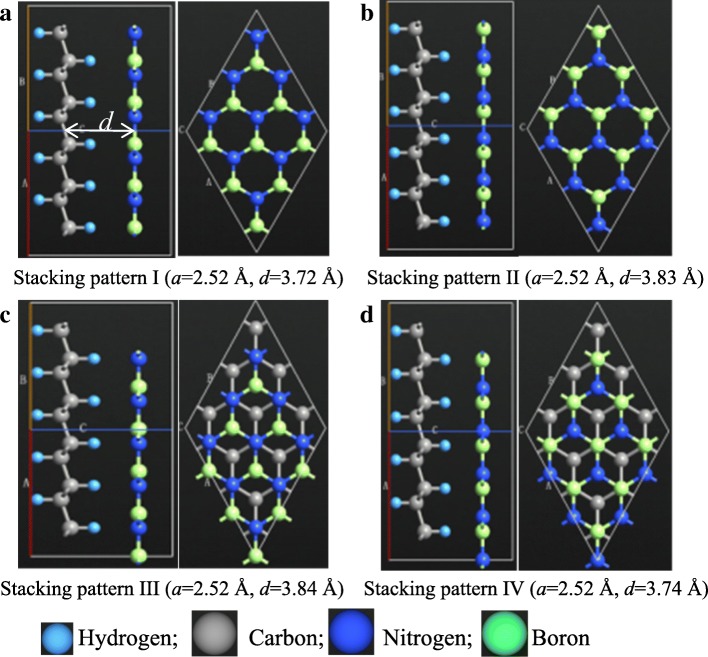
**a**–**d** Possible stacking patterns of 100%-HG/h-BN heterobilayer

Firstly, different stacking patterns between h-BN and HG were investigated, where the HG is 100% hydrogenated. It should be emphasized that the interaction between HG and h-BN is vdW force, which is far weaker than covalent bond. Hence, it is unnecessary to analyze the other HG/h-BN heterobilayers. There are four possible stacking patterns for the heterobilayer, as seen in Fig. 1a–d, where “*a*” is lattice parameter and “*d*” is interlayer distance. The interlayer distance is defined as the distance between the geometrical centers of HG layer and h-BN layer, as marked in Fig. 1a. In patterns I and II, the two skeletons are in AA stacking, while in patterns III and IV are in AB stacking. The structures were geometry optimized by the LBFGS optimizer method firstly. The convergence criteria for force tolerance are less than 0.001 eV/Å. After geometry optimization, the unit cell parameter is 2.52 Å for all the stacking patterns, while the interlayer distance depends on the stacking pattern. The interlayer distance of pattern I is the lowest, and pattern III is the highest. The vdW corrections of the four patterns are − 651.69 meV, − 658.14 meV, − 658.22 meV, and − 651.54 meV, respectively. Obviously, the tendency of vdW interaction coincides with that of the interlayer distance.

**Fig. 2:**
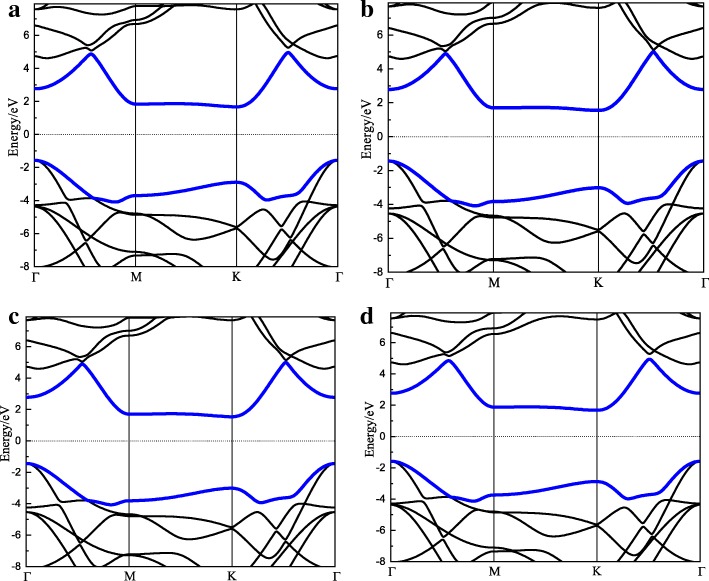
**a**–**d** The band structures of 100%-HG/h-BN heterobilayer stacking patterns I–IV

Band structure is one of the most important electronic properties. The corresponding band structures of stacking patterns I–IV are shown in Fig. 2. The two bold lines in each figure represent the bands including CBM (up) and VBM (down), respectively. Γ (0,0,0), M (0,0.5,0), K (0.333,0.333,0) are the symmetry points in the Brillouin zone. The main band structure information, including direct band gap (DBG), indirect band gap (IBG), CBM, and VBM positions, should be noticed. Generally, the four patterns have similar band structures. For patterns I–IV, the CBM and VBM are at point K and Γ, respectively. Patterns I and IV have similar DBG (4.35 eV) and IBG (3.25 eV), while the DBG and IBG of patterns II and III are about 4.22 eV and 2.98 eV. By comparing their interlayer distance, it can be concluded that the stronger interlayer interaction leads to the wider band gap. It should be emphasized that the band structure of single layer h-BN is also calculated with PBE. The band gap of h-BN is 4.65 eV which agrees well with the value reported in [30]. Overall, the method is suitable for h-BN.

**Fig. 3:**
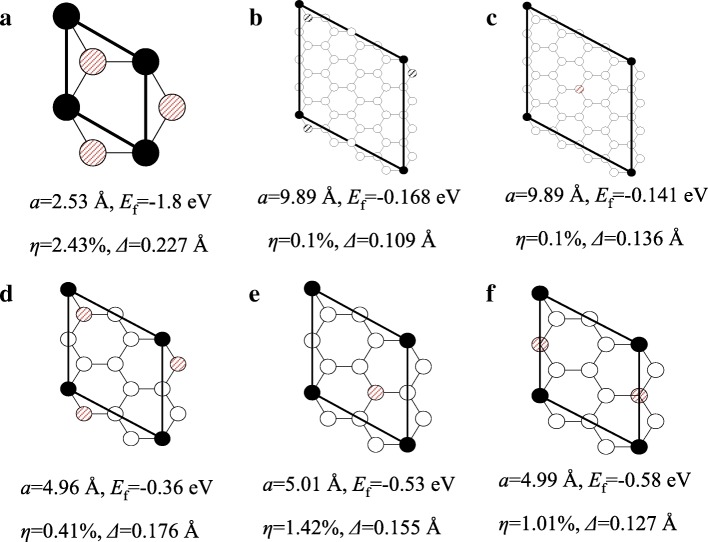
Schematic of primitive cell of HGs with different hydrogen coverage and pattern. **a** 100%. **b**, **c** 6.25% patterns I and II. **d**, **f** 25% pattern I–III

Secondly, the influences of hydrogen coverage and hydrogenation patterns are considered, whereas the influence of hydrogenation origins from the changing of covalent bonds, which is much stronger than vdW force. Hence, only HG monolayer is investigated in this part. The considered structures are shown in Fig. 3, where “” and “”denote the carbon atoms bonded with hydrogen atom at different sides. For the sake of the stability of the whole structure, hydrogen atoms are evenly distributed on each side. For 100% HG, it only has one stable pattern. Twenty-five percent HG composed by 8C and 2H has three different patterns. For 6.25% HG, it has 32C and 2H in the primitive cell. Only two patterns of 6.25% HG are considered. As shown in Fig. 3b, c, two hydrogenated carbon atoms are adjacent to each other in pattern I and away from each other in pattern II. It should be noticed that 6.25% pattern I, 25% pattern I and 100% HG are the same type (two hydrogenated carbon atoms are adjacent). In Fig. 3, *E*_f_ is the formation energy per atom

3 $$ {E}_{\mathrm{f}}=\frac{E_{\mathrm{total}}-{n}_{\mathrm{H}}{E}_{\mathrm{H}}\hbox{-} {E}_{\mathrm{graphene}}}{n_{\mathrm{H}}}, $$

where *E*_total_ is the total energy of HG, *E*_graphene_ refers to the energy of pristine graphene, *E*_H_ is the energy per atom of the H_2_ molecule, and *n*_H_ is the number of the adsorbed hydrogen atoms. *E*_f_ is used to check the stability of the structure, and the negative *E*_f_ suggests thermodynamics stability. The results in Fig. 3 imply that all the listed HGs are stable. *η* denotes the percentage rise of the lattice parameter of HG in contrast to graphene (the minimum unit cell length of graphene is 2.47 Å). On the whole, the lattice enhancement decreases with the decreasing hydrogen coverage. For 6.25% HG, *η* is almost negligible. Besides the hydrogen coverage, hydrogenation pattern also influences the lattice. For 25% HG, pattern I is enlarged least among the three patterns, mainly because the hydrogenated carbon atoms are adjacent. *Δ* is the buckling parameter, which is defined as the standard deviation of the out-of-plane displacements of the carbon atoms. Generally, the buckling parameter increases with the increased hydrogen coverage.

**Fig. 4:**
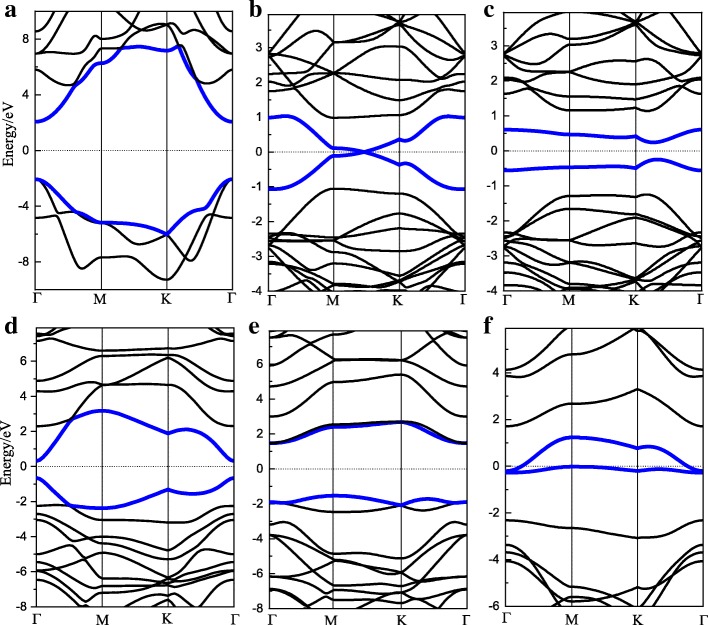
Band structures of HGs. **a** 100%. **b**, **c** 6.25% pattern I and II. **d**, **f** 25% pattern I–III

The band structures of the above HGs are shown in Fig. 4. The band gap of 100% HG is about 4.14 eV, in good agreement with the previous literature [16, 31]. For 25% HG, the band gap is strongly affected by the hydrogenation pattern. Pattern II has an IBG of 3.0 eV, while the IBG of pattern III is 0 eV. The IBG from zero to nonzero indicates a transition from metallic to semiconductor. In addition, pattern II has different DBG and IBG, suggesting that its CBM and VBM are at different points. For 6.25% HG, the VBM and CBM are at the same points for both of the two patterns, which of pattern I is (0.153, 0.423, 0) and pattern II is (0.24, 0.24, 0). The band gap of two 6.25% HGs are 0 eV and 0.49 eV, both of which reduced significantly in contrast to that of 100% HG. Generally, both hydrogen coverage and hydrogenation patterns are effective methods to modulate band gap.

Table 1 presents the estimated values of elastic modulus *C*_2D_, effective mass m^*^ and deformation potential constant *E*_1_. *C*_2D_ and m^*^ are direction-dependent parameters. Among all the directions, ΓM and ΓK are the most concerned. Hence, *C*_2D_ (ΓM/ΓK) and m* (ΓM/ΓK) are listed in Table 1. *C*_2D_ = *ρv*_g_^2^, where *ρ* is the density and *v*_g_ denotes group velocity of acoustic phonon. Because hydrogenation has few effects on group velocity, *C*_2D_ of different HGs are similar with each other. The HG *v*_g_ is about 23 km/s in ΓK direction and 19.4 km/s in ΓM, so *C*_2D_ (ΓK) is much higher than *C*_2D_ (ΓM). The deformation potential constant has no regular tendency with the different patterns. Generally, the vdW interaction between HG and h-BN increases the deformation potential constant.

**Table 1:** The elastic modulus *C*_2D_, effective mass m^*^, and deformation potential constant *E*_1_. “m_0_” denotes the electron mass 9.109 × 10^−31^ kg

Type	Pattern	*C*_2D_, N/m		m*, m_0_		*E*_1_, eV
		(ΓM)	(ΓK)	(ΓM)	(ΓK)	
Electron	100%	350.59	475.98	0.967	0.967	9.16
	25% pattern I	337.43	500.41	0.468	0.087	8.22
	25% pattern II	333.96	504.63	1.609	1.096	7.91
	25% pattern III	339.15	504.63	1.941	0.545	7.49
	6.25% pattern I	335.42	502.06	0.491	0.055	7.1
	6.25% pattern II	335.67	502.06	0.169	0.215	6.76
	Stacking pattern I	338.80	459.97	0.881	0.881	10.5
	Stacking pattern II	338.80	459.97	0.901	0.901	9.81
	Stacking pattern III	338.80	459.97	0.884	0.884	9.92
	Stacking pattern IV	338.80	459.97	0.905	0.905	9.45
Hole	100%	350.59	475.98	0.627	0.703	9.40
	25% pattern I	337.43	500.41	2.851	0.101	8.15
	25% pattern II	333.96	504.63	1.071	1.884	8.2
	25% pattern III	339.15	504.63	5.201	2.356	8.1
	6.25% pattern I	335.42	502.06	0.057	0.495	7.72
	6.25% pattern II	335.67	502.06	0.179	0.219	7.55
	Stacking pattern I	338.80	459.97	0.62	0.641	10.5
	Stacking pattern II	338.80	459.97	0.62	0.714	9.81
	Stacking pattern III	338.80	459.97	0.259	0.294	9.92
	Stacking pattern IV	338.80	459.97	0.26	0.282	9.45

Effective mass is more complicated, since it depends on carrier and direction. There are three points that should be noted on effective mass. First, the electron effective mass of 100% HG and 100%-HG/h-BN heterobilayer are isotropic, i.e., m*(ΓM) = m*(ΓK). The heterobilayer structure leads to a slight drop of electron effective mass compared with 100% HG monolayer. The stacking pattern has slight influence on the electron effective mass (all of the four stacking patterns are about 0.90). Second, under the same hydrogenation pattern (i.e., 100%, 25% pattern I and 6.25% pattern I), the electron m*(ΓK) decreases with the decreased hydrogen coverage. It is shown that the limit is 0.024 (the effective mass of graphene) as the hydrogen coverage reduces to zero. Third, under the same hydrogen coverage, effective mass is also affected by hydrogenation pattern. For 25% HG, the electron effective mass of pattern I is much lower than the other two. In a word, the effective mass is more likely to be affected by hydrogenation but not the elastic modulus and deformation potential constant.

In Table 2, the electron and hole mobilities are computed based on the above parameters. Because the effective mass is more likely to be affected, the tendency of mobility is similar with that of effective mass. Generally speaking, hydrogenation dramatically reduces the mobility of graphene. The theoretical mobility of graphene (3.2 × 10^5^ cm^2^/(V s)[28]) is several orders of magnitude higher than that of HG. In addition, HGs have asymmetric (μ_e_ ≠ μ_h_) and anisotropic (*μ*(ΓM) ≠ *μ* (ΓK)) mobilities. There are three details that should be noticed. First, under the same hydrogenation pattern, the electron mobility monotonously decreases with the increasing hydrogen coverage. But, if under different hydrogenation pattern, the conclusion is not always established. For example, the mobilities of 25% pattern II are lower than that of 100% HG. Second, for 25% and 6.25% HGs, pattern I has a higher *μ*_e_ compared to the other patterns. The *μ*_e_ (ΓK) of 25% pattern I is 8985.85 cm^2^/(V s) and of 6.25% pattern I is 23,470.98 cm^2^/(V s), much higher than black phosphorene [24] and MoS_2_ [32]. Third, the h-BN substrate affects the hole mobilities significantly, while it has little effect on the electron mobilities. It indicates the hole mobilities of stacking patterns I and II are close to that of HG monolayer, but much lower than that of stacking patterns III and IV. Hence, different stacking patterns have significant effects on hole mobilities but little effects on electron mobilities.

**Table 2:** The electron and hole mobilities in ΓM and ΓK direction. The subscripts “e” and “h” refer to electron and hole, respectively. Unit is cm^2^/(V s)

	*μ*_e_ (ΓM)	*μ*_h_ (ΓM)	*μ*_e_ (ΓK)	*μ*_h_ (ΓK)
100%	95.18	162.71	129.22	197.02
25% pattern I	1126.39	70.73	8985.85	2960.80
25% pattern II	53.21	69.54	118.03	59.73
25% pattern III	64.50	6.05	341.81	19.86
6.25% pattern I	1756.48	12,520.27	23,470.98	2158.01
6.25% pattern II	4856.81	3539.15	5710.03	4326.59
Stacking pattern I	84.33	167.47	114.49	219.91
Stacking pattern II	92.37	181.78	125.41	214.30
Stacking pattern III	93.84	1026.06	127.40	1227.19
Stacking pattern IV	98.66	1147.82	133.95	1436.75

**Fig. 5:**
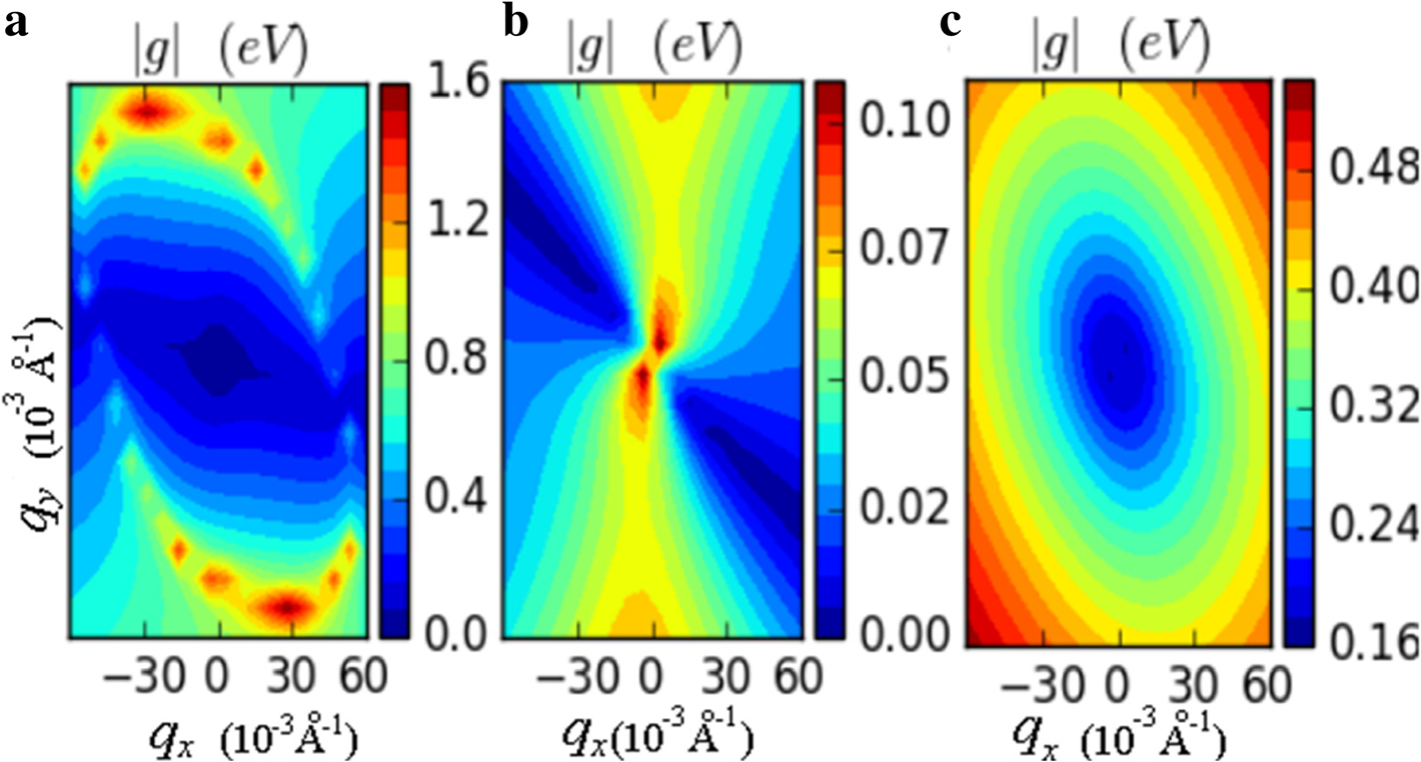
The electron-phonon interaction matrix elements |*g*| of **a** LA, **b** TA, and **c** ZA phonons

Moreover, the mobility of 100% HG was recalculated by considering all the electron-phonon interactions, namely longitude acoustic (LA), transverse acoustic (TA) and ZA phonons. The results show that the electron mobility is 105 cm^2^/(V s) in ΓK direction. Figure 5 gives the electron-phonon interaction matrix elements |*g*| of LA, TA and ZA phonons. It shows that the LA phonons dominate in electron-phonon interactions. On the whole, LA phonons have larger interaction strength with electrons compared with the TA and ZA phonons. Although the mobility value is slightly lower than that calculated by DPA method, the difference of two methods in HG is much less than that in arsenene, antimonene, and silicene. Generally, the DPA method is feasible in our study.

## Conclusions

In summary, the carrier mobilities of HG/h-BN heterobilayer were investigated based on the first-principles calculations in this paper. The influence on mobilities is discussed in terms of the stacking patterns of HG/h-BN heterobilayer, hydrogen coverage, and hydrogenation pattern. The elastic modulus *C*_2D_, effective mass m^*^, and deformation potential constant *E*_1_ are calculated to analyze the mobilities. The deformation potential constant has no regular tendency with the different patterns. The elastic modulus and the effective mass in HGs are direction-dependent. The results show that ΓK direction has a higher elastic modulus. The effective mass is more likely to be affected by different hydrogenations and stacking patterns. Under the same hydrogenation pattern, the electron mobility monotonously decreases with the increasing hydrogen coverage. Under the same hydrogen coverage, different patterns lead to a significant change of mobilities. For 25% and 6.25% HGs, the μ_e_ (ΓK) of 25% pattern I is 8985.85 cm^2^/(V s) and of the *μ*_e_ (ΓK) 6.25% pattern I is 23,470.98 cm^2^/(V s); both are much higher than the other patterns. As for the influence of h-BN substrate, different stacking patterns affect the hole mobilities significantly, but hardly affect the electron mobilities. The hole mobilities of stacking patterns I and II are close to that of HG monolayer, but much lower than that of stacking patterns III and IV. Overall, HG/h-BN heterobilayer has a considerable carrier mobility and band gap under a specific hydrogenation pattern, which has promising application prospects in electronics and photonics.

## Abbreviations

ATK: Atomistix ToolKit
CBM: Conduction band minimum
DBG: Direct band gap
DFT: Density functional theory
DPA: Deformation potential approximation
FET: Field-effect transistor
GGA: Generalized gradient approximation
h-BN: Hexagonal boron nitride
HG: Hydrogenated graphene
IBG: Indirect band gap
PBE: Perdew-Burke-Ernzerhof
VBM: Valence band maximum
vdW: van der Waals
